# Tissue-specific impact of *FADS* cluster variants on *FADS1* and *FADS2* gene expression

**DOI:** 10.1371/journal.pone.0194610

**Published:** 2018-03-28

**Authors:** Lindsay M. Reynolds, Timothy D. Howard, Ingo Ruczinski, Kanika Kanchan, Michael C. Seeds, Rasika A. Mathias, Floyd H. Chilton

**Affiliations:** 1 Department of Epidemiology & Prevention, Wake Forest School of Medicine, Winston-Salem, North Carolina, United States of America; 2 Department of Biochemistry, Wake Forest School of Medicine, Winston-Salem, North Carolina, United States of America; 3 Division of Allergy and Clinical Immunology, Department of Medicine, Johns Hopkins University, Baltimore, Maryland, United States of America; 4 Department of Internal Medicine/Section on Molecular Medicine, Wake Forest School of Medicine, Winston-Salem, North Carolina, United States of America; 5 Department of Physiology and Pharmacology, Wake Forest School of Medicine, Winston-Salem, North Carolina, United States of America; Universitat de Lleida, SPAIN

## Abstract

Omega-6 (n-6) and omega-3 (n-3) long (≥ 20 carbon) chain polyunsaturated fatty acids (LC-PUFAs) play a critical role in human health and disease. Biosynthesis of LC-PUFAs from dietary 18 carbon PUFAs in tissues such as the liver is highly associated with genetic variation within the fatty acid desaturase (*FADS)* gene cluster, containing *FADS1* and *FADS2* that encode the rate-limiting desaturation enzymes in the LC-PUFA biosynthesis pathway. However, the molecular mechanisms by which *FADS* genetic variants affect LC-PUFA biosynthesis, and in which tissues, are unclear. The current study examined associations between common single nucleotide polymorphisms (SNPs) within the *FADS* gene cluster and *FADS1* and *FADS2* gene expression in 44 different human tissues (sample sizes ranging 70–361) from the Genotype-Tissue Expression (GTEx) Project. *FADS1* and *FADS2* expression were detected in all 44 tissues. Significant cis-eQTLs (within 1 megabase of each gene, False Discovery Rate, FDR<0.05, as defined by GTEx) were identified in 12 tissues for *FADS1* gene expression and 23 tissues for *FADS2* gene expression. Six tissues had significant (FDR< 0.05) eQTLs associated with both *FADS1* and *FADS2* (including artery, esophagus, heart, muscle, nerve, and thyroid). Interestingly, the identified eQTLs were consistently found to be associated in opposite directions for *FADS1* and *FADS2* expression. Taken together, findings from this study suggest common SNPs within the *FADS* gene cluster impact the transcription of *FADS1* and *FADS2* in numerous tissues and raise important questions about how the inverse expression of these two genes impact intermediate molecular (such a LC-PUFA and LC-PUFA-containing glycerolipid levels) and ultimately clinical phenotypes associated with inflammatory diseases and brain health.

## Introduction

Landmark studies by Burr and Burr almost 100 years ago demonstrated that dietary eighteen carbon (18C-) polyunsaturated fatty acids (PUFAs), linoleic acid (LA, 18:2n-6) and alpha-linolenic (ALA, 18:3n-3), are essential to health [1, 2]. Later it was discovered that these 18C-PUFAs are substrates that enter omega-6 (n-6) and omega-3 (n-3) long chain (LC-) PUFA biosynthetic pathways resulting in the formation of biologically-active LC-PUFAs. In two parallel and competing pathways, desaturase and elongase enzymes convert n-6 or n-3 PUFAs into several LC-PUFAs. In the n-6 arm of the pathway, arachidonic acid (ARA, 20:4n-6) is synthesized from LA, utilizing 2 desaturation and 1 elongation enzymatic steps encoded by *FADS2*, *FADS1* and *ELOVL5*, respectively [3–5]. The n-3 LC-PUFA, eicosapentaenoic acid (EPA, 20:5n-3) is synthesized from ALA employing the same three enzymatic steps. Twenty-two carbon n-6 and n-3 LC-PUFAs, adrenic acid (ADA, 22:4n-6) and docosapentaenoic acid (DPA, 22:5n-3) can then be formed utilizing an additional elongation step (encoded by *ELOVL2*), and docosahexaenoic acid (DHA, 22:6n-3) synthesized from DPA with another desaturation step (encoded by *FADS2*) or three additional biosynthetic steps (2 elongation, 1 desaturation and 1 β-oxidation) [6–9]. Once formed, LC-PUFAs are transported to cells and tissues in circulation as free fatty acids bound to albumin or esterified to complex lipids such as phospholipids (PL), cholesterol esters, and triglycerides in lipoprotein particles [10].

Until recently, it was assumed that the metabolic capacity of the LC-PUFA biosynthetic pathway was fairly uniform for all humans. This premise was supported by stable isotope, metabolic studies carried out in European ancestry populations, which indicated that small proportions of ingested dietary 18-carbon PUFAs are converted into LC-PUFAs [11]. The desaturase enzymes within the pathway, encoded by the three genes (*FADS1*, *FADS2*, and *FADS3*) in the *FADS* gene cluster region (chr11:61,540,615–61,664,170, hg19) have long been recognized as the rate-limiting steps in the conversion of 18C-PUFAs to LC-PUFAs [3]. Studies over the past decade have challenged the concept that biosynthesis of LC-PUFAs is uniform among individuals and populations by demonstrating that common genetic and epigenetic variation in and near *FADS1* and *FADS2* are strongly associated with the levels of LC-PUFAs found in circulation, blood cells, and tissues [4, 12–21]. Moreover, the frequency of these variants and thus the capacity to synthesize LC-PUFAs can differ dramatically across diverse human populations [15, 16]. In addition to LC-PUFA levels themselves, genetic variation in the *FADS* cluster is associated with numerous human phenotypes, including inflammatory [22] and cardiovascular disorders [21, 23], blood lipid levels including low-density lipoprotein (LDL) and triglyceride levels [17, 24–27], coronary artery disease [23, 28, 29], insulin resistance [30], perinatal depression [31], atopic diseases [32–34], attention deficit disorder/hyperactivity, intelligence and memory in children [35, 36]. Taken together, these findings support a relationship between genetic variations in the *FADS* gene cluster and several human diseases that may involve tissue-specific levels of LC-PUFAs.

It has also been generally presumed that the liver is the primary organ responsible for the biosynthesis of LC-PUFAs that are released into circulation and eventually incorporated into tissues. However, other cells and tissues are known to have their own endogenous capacity to synthesize LC-PUFAs from dietary 18C-PUFAs [37–39]. While associations between genetic variants in *FADS1* and *FADS2* and LC-PUFA levels, as well as desaturase activities and certain phenotypes have been established, little is known about the range of tissues most capable of LC-PUFA biosynthesis and the molecular mechanisms by which these *FADS* polymorphisms exert their effects. Expression quantitative trait loci (eQTL) mapping is a powerful approach to examine specific genetic variation(s) associations with gene expression levels, but eQTLs are typically examined in only a few accessible tissues. This creates challenges in understanding how genetic variants may influence gene expression levels and in this case LC-PUFA biosynthesis across a wide range of tissues. The Genotype-Tissue Expression (GTEx) Project was designed to address many of these limitations by providing a platform to examine the relationships between genetic variation and gene expression across a wide range of human tissues [40]. The current study has leveraged the publicly available GTEx data to better understand which tissues have the capacity to express *FADS* cluster genes and to better understand the potential for tissue-specific influences of genetic variation in the *FADS* cluster on *FADS* gene expression and PUFA desaturation, by examining the associations between *FADS* cluster single nucleotide polymorphisms (SNPs) and *FADS1* and *FADS2* expression in 44 tissues.

## Methods

### *FADS* cluster gene expression and eQTL analysis in GTEx

The GTEx Project [40] data portal was used to investigate *FADS1* and *FADS2* gene expression across human tissues and genetic influences on *FADS1* and *FADS2* expression across 44 tissues (data source: GTEx data version V6p). As described previously, GTEx data includes gene expression measured in various tissues collected during autopsies within 24 hours of death for 572 individuals (34.4% female; 84.3% White, 13.7% African American, 1% Asian, 1% not reported). GTEx version V6p eQTL analysis utilized gene expression measured using RNA-sequencing (Illumina TrueSeq) for samples with an RNA Integrity Number (RIN, as measured by Agilent Bioanalyzer) of 6.0 or higher. GTEx performed the following quality control measures for all results accessed through the data portal. Gene expression levels passed the quality control thresholds of >0.1 reads per kilobase of transcript per million (RPKM) in at least 10 individuals and ≥6 reads in at least 10 individuals. Expression values were quantile normalized to the average empirical distribution across samples. For each gene, expression values were inverse quantile normalized to a standard normal distribution across samples. Outliers were identified and excluded using a correlation-based statistic similar to methods previously described [41]. GTEx genotyped single nucleotide polymorphisms (SNPs) using whole genome sequencing (HiSeq X; first batch on HiSeq 2000), whole exome sequencing (Agilent or ICE target capture, HiSeq 2000), or microarray (Illumina OMNI 5M Array or 2.5M SNP Array, or Illumina Human Exome SNP Array) from blood samples, and imputed using 1000 Genomes Project Phase I, version 3 for 449 donors. A call rate threshold of 95% and a minor allele frequency (MAF) > = 1% (a tissue specific cutoff, as sample sets vary by tissue) was used.

Publicly available eQTL results and trait expression levels were downloaded from the GTEx Project data portal [40]. Briefly, GTEx generated the association results using linear regression, associating normalized log-transformed gene expression levels with SNPs. A threshold of ≥ 70 samples per tissue was used for eQTL analysis, resulting in testing of 44 tissues for eQTL analysis. Association analyses were adjusted for sex, genotyping platform, and the top three genotyping-based principal components to adjust for potential population stratification by race/ethnicity background. The effect size of the eQTLs was defined as the slope of the linear regression, comparing the alternative allele (ALT) to the reference allele (REF) in the human genome reference GRCh37/hg19. GTEx used a false discovery rate (FDR) < 0.05 to correct for multiple hypothesis testing [42]. This paper includes the publicly available GTEx eQTL results from 7,161 SNPs located within one megabase of *FADS1* and *FADS2* transcription start sites.

### *In silico* functional analysis

RegulomeDB [43] version 1.1 was used to prioritize the *FADS* cluster eQTLs identified by GTEx data, based on predicted function. RegulomeDB *in silico* prediction utilizes known and predicted DNA regulatory elements from public datasets including the NCBI Gene Expression Omnibus (GEO), the ENCODE project [44], and published literature. Regulatory regions predictions are based on previously identified overlapping features characterized to regulate gene expression, including DNAase hypersensitive sites, transcription factor binding sites, and promoter/enhancer regions.

## Results

### *FADS1* and *FADS2* gene expression

**Fig 1:**
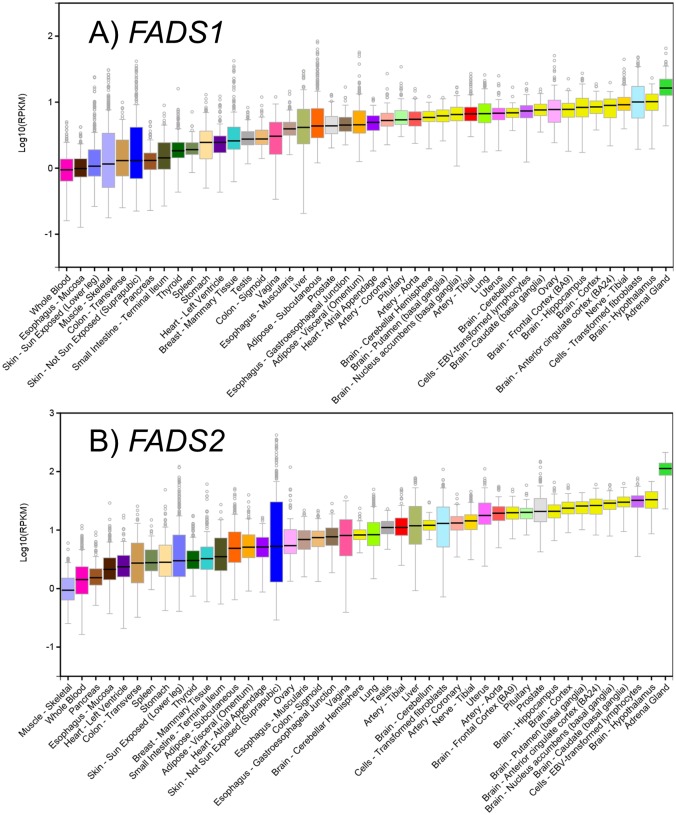
*FADS1* and *FADS2* expression across 44 human tissues. Boxplots of *FADS1* (top) and *FADS2* (bottom) mRNA expression (log_10_RPKM—y axis) are shown across tissues (x-axis) from GTEx; outliers are shown as circles; median expression is represented as the center line.

*FADS1* and *FADS2* mRNA expression were detected (at an expression threshold of >0.1 RPKM in at least 10 individuals and ≥6 reads in at least 10 individuals) in all 44 human tissues found in GTEx (Fig 1). Median gene expression levels across tissues were highest in the adrenal gland and brain tissues for both *FADS1* and *FADS2*. Overall, *FADS2* was expressed at higher median levels than *FADS1*, with *FADS1* ranging from 0.89 RPKM in whole blood to 16.2 RPKM in adrenal gland tissue, and *FADS2* ranging from 0.89 RPKM in skeletal muscle to 112.7 RPKM in adrenal gland tissue.

### *FADS1* and *FADS2* eQTL analysis

**Fig 2:**
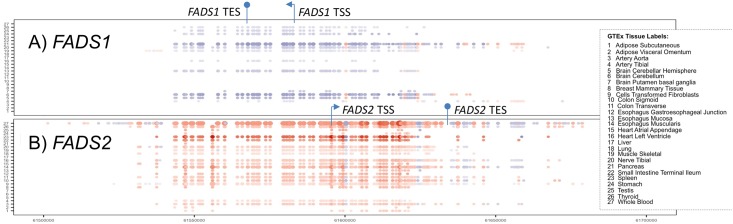
*FADS1* and *FADS2* cis-eQTLs. Significant eQTLS (FDR<0.05) associated with gene expression of *FADS1* (A) or *FADS2* (B) identified in 27 human tissues from GTEx. The significance of the detected association is represented as the size of the circle (larger the circle, more significant the result), and the color represents the direction of effect of the alternate (alt) allele (red for increased transcription with alt allele, blue for decreased transcription). *FADS1* and *FADS2* gene transcription start sites (TSS) and transcription end sites (TES) illustrate the location of FADS1 and FADS2 genes relative to the cis-eQTLs.

Overall, 27 of the 44 tissues from GTEx contained significant cis-eQTLs for either *FADS1* or *FADS2* after adjusting for multiple testing (FDR<0.05), as depicted in Fig 2. For *FADS1*, 167 SNPs were identified as significant (FDR<0.05) cis-eQTLs, which were found in at least one of 12 tissues, including artery (tibial), brain (cerebellar hemisphere, cerebellum, putamen basal ganglia), esophagus (Mucosa), heart (left ventricle), muscle (skeletal), nerve (tibial), pancreas, stomach, testis, and thyroid tissues. For *FADS2*, 233 SNPs were identified as significant (FDR<0.05) cis-eQTLs, which were identified in at least one of 23 tissues, including adipose (subcutaneous, visceral omentum), artery (aorta, tibial), breast (mammary tissue), transformed fibroblasts, colon (sigmoid, transverse), esophagus (gastroesophageal junction, mucosa, muscularis), heart (atrial appendage, left ventricle), lung, muscle (skeletal), nerve (tibial), pancreas, small intestine (terminal ileum), spleen, stomach, testis, thyroid, and whole blood. The 233 SNPs found to be *FADS2* eQTLs included 146 SNPs that were also *FADS1* eQTLs, of which 73 SNPs were significantly (FDR<0.05, p≤ 8.30x10^-5^) associated with both expression of *FADS1* and *FADS2* in same tissue (in at least one of the six following tissues: artery (tibial), esophagus (mucosa), heart (left ventricle), muscle (skeletal), nerve (tibial), and thyroid, see S1 Table). Surprisingly, all of these SNPs were associated with *FADS1* and *FADS2* expression in opposite directions as depicted by the blue and red colors in Fig 2.

### *In silico* predicted functional effects of *FADS1* and *FADS2* eQTLs

Identification of predicted regulatory features for gene expression (from ENCODE[44] and RegulomeDB [43]) such as promoters, enhancers, insulators, DNase hypersensitive regions, and transcription factor binding sites (TFBS) overlapping the identified eQTLs provided additional evidence supporting the potential functional effects of ~30 of the 73 eQTLs for both *FADS1* and *FADS2*. (S2 Table and S1 Fig). High linkage disequilibrium (LD) was observed between most of these potentially functional variants (r^2^≥0.8), as shown in S1 Fig. Five of the predicted functional eQTLs were not in high LD with other eQTLs (rs61896141, rs968567, rs61897793, rs174575, and rs174627). Six of the potential functional SNPs were previously identified as eQTLs for *FADS2* in monocytes (rs174534, rs174547, rs174549, rs174548, rs174583, and rs174577) [45]. All six of these SNPs overlap transcription factor binding sites, three of which also lie in DNase hypersensitive regions (rs174534, rs174547, and rs174583). These six SNPs are in moderate to high LD (ranging from r^2^ = 0.58 between rs174548 and rs174534 to r^2^ = 0.97 between rs174548 and rs174549). Only one of these SNPs (rs174548) was previously annotated as a *FADS1* eQTL (in liver cells [46]) in RegulomeDB.

Rs174548 (chr 11:61,571,348, hg19) was chosen as a representative candidate functional eQTL for *FADS1* and *FADS2* for a number of reasons, including that it was among the most significant eQTLs for *FADS1* and *FADS2* in GTEx. Additionally, rs174548 had a RegulomeDB score of 1f, indicating that it was likely to affect protein binding and was previously linked to expression of genes, including *FADS1* (in liver [46]), *FADS2* (in monocytes [45]), and *FADS3* (in liver [46]). Rs174548 also overlaps a binding site for RNA polymerase II subunit A (POLR2A in B cell line) and has been previously demonstrated to be associated with numerous traits, including plasma levels of dihomogamma-linolenic acid, DGLA, 20:3n-6, p = 7 x 10^−31^ [47], blood levels of ARA (p = 1 x 10^−84^) [48], triglycerides (p = 5 x 10^−14^) [49], high density lipoprotein (HDL) cholesterol levels (p = 1 x 10^−12^) [50], and ratios of ARA/DGLA (p = 2 x 10^−361^) in blood [48].

**Fig 3:**
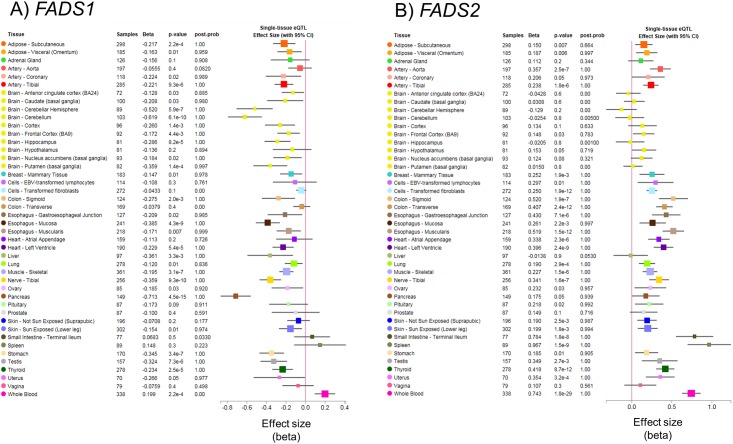
rs174548 genotype associations with *FADS1* and *FADS2* across tissues. Nominal associations (effect size and 95% confidence interval) between rs174548 genotypes (G, alt allele vs C, ref allele) with *FADS1* (A) and *FADS2* (B) are shown across GTEx tissues. The effect size of the eQTLs was defined as the slope of the linear regression, comparing the alt allele (G) to the reference allele (C).

Similar to other potentially functional SNPs in high LD (such as rs174560, r^2^ = 0.99), rs174548 was associated with *FADS1* and *FADS2* expression with opposite effect directions in numerous tissues. Fig 3 illustrates the effect size and 95% confidence intervals for the rs174548 eQTL (alternative allele G, reference allele C) across tissues for *FADS1* and *FADS2*. The strongest associations between rs174548 and *FADS1* were identified in pancreas (beta = -0.71, p = 4.5x10^-15^) and brain tissue (beta = -0.62, p = 6.1x10^-10^), with lower *FADS1* expression associated with increased copies of the minor allele (G, minor allele frequency = 0.30 in HAPMAP CEU). Notably, unlike brain tissue, *FADS1* was not highly expressed in pancreas tissue (Fig 1). Increased copies of the rs174548 G allele was also nominally (p<0.05) associated with lower *FADS1* expression in tissues thought to be particularly relevant to LC-PUFA biosynthesis, including adipose (subcutaneous, beta = -0.22, p = 2.2x10^-4^) and liver (beta = -0.36, p = 3.3x10^-3^). In contrast, the rs174548 G allele was nominally associated with higher *FADS2* expression in adipose tissue (subcutaneous, beta = 0.15, p = 7x10^-3^), but was not significantly associated with *FADS2* expression in brain or liver. The rs174548 G allele was also associated with increasing *FADS2* expression in other tissues, including artery, breast, colon, esophagus, heart, lung, muscle, nerve, ovary, skin (sun exposed), stomach, testis, and thyroid tissues. Of note, the only tissue with similar effect directions observed for rs174548 associations with *FADS1* and *FADS2* was whole blood, where the G allele was associated with higher expression of both *FADS1* and *FADS2*; however, neither *FADS1* nor *FADS2* were highly expressed in whole blood (Fig 1). Also, although the association between rs174548 and *FADS1* was nominally significant in tissues such as whole blood and subcutaneous adipose tissue, the associations did not reach the multiple testing correction threshold (FDR<0.05, p≤ 8.30x10^-5^).

**Fig 4:**
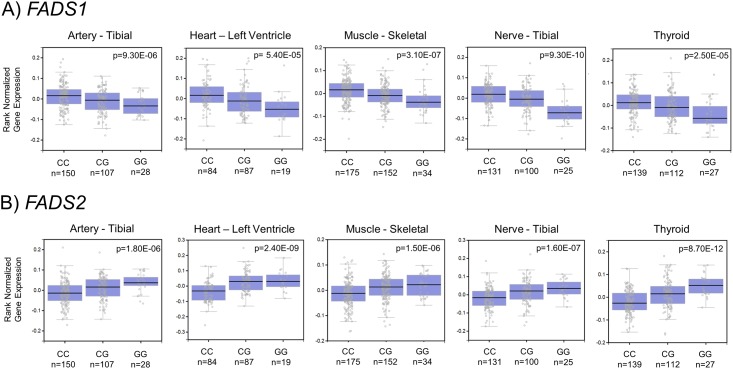
*FADS1* and *FADS2* expression by rs174548 genotype. *FADS1* (A) *FADS2* (B) rank normalized gene expression (Y-axis) by rs174548 genotype (G: alt allele, C: ref allele) in the five tissues with significant (FDR<0.05) associations between rs174548 and *FADS1* and *FADS2* in the same tissues.

Associations between rs174548 and both *FADS1* and *FADS2* were significant at the multiple testing correction threshold (FDR<0.05, p≤ 8.30x10^-5^) in five tissues. Specifically, the G allele of rs174548 was associated with lower expression of *FADS1* and higher expression of *FADS2* in all five tissues. Tissues included artery (tibial), heart (left ventricle), muscle (skeletal), nerve (tibial), and thyroid (Fig 4 and S3 Table).

## Discussion

Candidate gene-based studies and GWAS have revealed that SNPs throughout the *FADS* cluster are associated with LC-PUFA levels, 18C-PUFA to LC-PUFA conversion efficiencies, as well as numerous disease-related molecular phenotypes and the risk of human diseases [5, 21, 22, 28–30, 47–49, 51, 52]. However, determining causative variants, molecular mechanisms, and relevant tissues contributing to these associations has been difficult, due in large part to extensive LD throughout the *FADS* cluster and the capacity of numerous tissues to synthesize LC-PUFAs. The liver has long been assumed to be the primary tissue that synthesizes LC-PUFAs found in circulation. We and others have demonstrated that in the liver, several genetic and epigenetic variants within and near the *FADS* cluster are associated with LC-PUFA levels [53, 54]. Wang and colleagues demonstrated that six SNPs within the *FADS* cluster (*FADS1*, *FADS2*, and *FADS3*) were consistently and significantly associated with *FADS1* gene expression in liver, but not with expression of *FADS2* or *FADS3*, with rs174556 exerting the highest correlation[50]. Pan and colleagues systematically tested SNPs in putative regulatory regions in the *FADS* cluster using a luciferase reporter assay and found a SNP in *FADS1* (rs174557) in an *Alu* element that they postulated affects *FADS1* enhancer activity in a liver cell line (HepG2) by altering competitive transcription factor binding between the suppressive PATZ1 and the activating complex of SP1/SREBP1c [55]. These studies suggest that *FADS1* gene expression plays a causal role in the previously observed associations between *FADS* cluster SNPs and LC-PUFA biosynthesis. While these studies are clearly important initial steps in determining putative regulatory SNPs that impact *FADS1* expression, they are limited by the fact that they have not examined these eQTLs across a wide range of tissues.

To better clarify the relationship between genetic polymorphisms identified by GWAS and disease etiology, GTEx has investigated the relationships between genetic polymorphisms and gene expression in several organs and tissues. The current study utilized publicly available results generated by GTEx to examine the relationship between genetic variation within or near the *FADS* cluster and *FADS1* and *FADS2* gene expression in 44 tissues. There were several interesting and surprising observations from this analysis. As expected, the levels of *FADS1* and *FADS2* expression, and the association of *FADS* cluster SNPs, varied greatly among different organs and tissues. While all tissues examined demonstrated *FADS1* and *FADS2* expression, significant cis-eQTLs for *FADS1* or *FADS2* were detected in 27 tissues, with several cis-eQTLs common to both *FADS1* and *FADS2* expression within the same tissue. The most unexpected result from this study was the observation that in tissues with common eQTLs for both *FADS1* and *FADS2*, the variant associations with expression of *FADS2* gene expression were in the opposite effect directions as the associations with *FADS1* gene expression.

Rs174548 was chosen as a candidate functional eQTL for *FADS1* and *FADS2* for many reasons, including its predicted functional score from RegulomeDB, and its previous identification as a *FADS1* eQTL in liver [46] and a *FADS2* eQTL in monocytes [45]. Additionally, it has strong associations with LC-PUFA biosynthetic capacity and other important molecular phenotypes [26, 47–49], and is among the most significant eQTLs for *FADS1* and *FADS2* in GTEx. Rs174548 is also in high LD with many nearby variants predicted to have functional effects on transcription factor binding (by RegulomeDB), including one of the most significant SNPs (rs174556, r^2^ ~ 0.80) previously associated with *FADS1* in liver [50], located ~10 kb away. In GTEx, rs174556 was also an eQTL for both *FADS1* and *FADS2* expression in multiple tissues (but only *FADS1* in liver). The other previously identified *FADS1* eQTL in liver (rs174557) that was reported to alter the activity of a SREBP1c inducible *FADS1* enhancer [55] was not among the variants captured in GTEx. However, rs174560, which was located on the same 646-bp luciferase construct as rs174557 [55], was among the 73 cis-eQTLs identified for both *FADS1* and *FADS2* in GTEx in the same tissues, and was in almost perfect LD (r^2^>0.99) with the candidate functional eQTL chosen here, rs174548.

In tissues thought to be particularly relevant to LC-PUFA biosynthesis such as the liver and brain, the minor allele of rs174548 (G) was associated with lower expression of *FADS1*, but was not associated with *FADS2*. The minor allele of rs174548 in subcutaneous adipose tissue was also associated with lower expression of *FADS1* but higher expression of *FADS2* (with nominal significance, not after multiple testing corrections). A similar pattern was observed in all five tissues (including tibial artery, heart left ventricle, skeletal muscle, tibial nerve, and thyroid tissues) that had significant associations after genome-wide multiple testing corrections between rs174548 and both *FADS1* and *FADS2*, where the minor allele of rs174548 was associated with lower expression of *FADS1* and higher expression of *FADS2*. The only exception to this pattern was the observation in whole blood, which did not reach genome-wide significance, where the minor allele of rs174548 was nominally associated with increased *FADS1* and *FADS2* expression. However, it is important to point out that whole blood had very low levels of *FADS1* and *FADS2* expression.

The inverse gene expression patterns of *FADS1* and *FADS2* with cis-eQTLs is intriguing as these two genes are situated in a “head-to-head” orientation, with intergenic regulatory regions (containing putative promoters and an enhancer) that may affect both genes. This regulatory landscape is relatively common in the genome, and it has been reported that approximately 10% of protein coding transcripts are encoded in this orientation [56–59]. Although expression of head-to-head genes is usually positively correlated, genome-wide analysis has shown that negatively correlated expression also occurs [58, 59]. For *FADS1* and *FADS2*, inverse expression may be due to steric hindrance caused by the binding of transcription factors targeting expression of one of the two genes. Given the importance of these genes in sequential steps in this PUFA biosynthetic pathway, it is also conceivable that the genes may alternate between positive and negative correlation, depending on the availability of their substrate. Additionally, allele-specific methylation analysis has revealed that SNPs in the *FADS* cluster of human liver tissue may interact with the DNA methylation in the common regulatory region between *FADS1* and *FADS2*, and such a mechanism could also impact gene expression [53, 60]. Future investigation will be necessary to better delineate the effects of genetic variation in the *FADS* gene cluster on the regulatory mechanisms that impact the expression of these two genes.

How does the inverse eQTL pattern for *FADS1* and *FADS2* affect our understanding of LC-PUFA biosynthesis and related phenotypes? Studies that combine GWAS and metabolomics (in serum or whole blood) have proven very powerful for identifying genes that participate in the synthesis of LC-PUFA and LC-PUFA complex lipids [61]. Certainly, when examining levels of circulating and liver 18C-PUFAs and LC-PUFAs, and precursor-product ratios of the *FADS1* and *FADS2* reactions, most studies point to the importance of polymorphisms near *FADS1*. For example, Geiger and colleagues showed that phospholipid metabolites with four double bonds (*i*.*e*., ARA) were associated with SNPs in *FADS1* [61–63] and this effect was observed for all major phospholipid species [61]. Additionally, the association with SNPs in *FADS1* increased up to 14-fold when examining ratios of phospholipids that contained *FADS1* precursors (DGLA) and products (ARA). The authors point out that this effect is so strong that “if the molecular function of *FADS1* had not been already known, the association between the SNP and the different glycerophospholipid concentrations would have allowed one to deduce its enzymatic activity.” Similarly, rs174548 (along with numerous other SNPs in high LD) is strongly associated with levels of ARA (p = 1 x 10^−84^), but the effect size is increased 4-fold when measuring the *FADS1* product to precursor ratio of ARA/DGLA (p = 2 x 10^−361^) in human blood [48]. A recent study indicated that *FADS1* polymorphisms play a key role in hepatic total fat content by modulating levels of LC-PUFA-containing phospholipids and *FADS1* expression [50].

Numerous tissues have their own endogenous capacity to synthesize LC-PUFAs, and this study shows that many of these same tissues have eQTLs inversely correlated for *FADS1* and *FADS2*. A major question that arises from this paper is how *FADS* cluster SNPs impact levels of LC-PUFAs or *FADS1* and *FADS2* precursor-product ratios in these tissues. We have recently examined prostate cancer tissues from patients undergoing radical prostatectomy and demonstrated that prostate cancer tissues have great capacity to synthesize LC-PUFAs from 18-C PUFAs[39]. The presence of the G allele at *FADS* SNP rs174537 was significantly associated with higher levels of both n-6 and n-3 LC-PUFAs. Importantly, rs174537 genotypes had much greater effects on the ratio of ARA to DGLA than the effects on ARA alone or precursor-product ratios of *FADS2* activity, suggesting that *FADS1* activity, expression, or both, are related to genotypic variation in the *FADS* cluster. However, few studies have examined the relationship between *FADS* cluster variants and LC-PUFA biosynthesis in other tissues that have endogenous capacities to synthesize LC-PUFAs. It remains unclear how alterations in *FADS1* and *FADS2* gene expression in difficult to ascertain tissues, such as those identified in this study including the artery, heart, muscle, nerve, and thyroid tissues, may influence LC-PUFA biosynthesis. More studies are necessary to understand the impact of *FADS* gene cluster eQTLs in inverse directions for *FADS1* and *FADS2* on LC-PUFA levels in these tissues.

Interpretation of the findings from this study is limited by the combined analysis of both sexes and Caucasian and African American populations. However, alleviating concerns that the *FADS1* and *FADS2* eQTL results from GTEx were driven by population stratification, all analyses included an adjustment for sex and the top three principal components to adjust for genetic ancestry. Additionally, rs174548 and other potentially functional eQTLs are common variants in both Caucasian and African American populations (MAF in a European population = 0.31 and MAF in an African population = 0.18, from 1000 genomes). Future studies with larger sample sizes will be necessary to confirm the opposite effects of increasing minor allele copies of rs174548 on *FADS1* and *FADS2* expression across sex- and ethnic-specific strata.

It is important to note that results of this study are limited to the associations between *FADS* cluster variants and gene expression of *FADS1* and *FADS2*. It remains unclear how the opposing relationships between *FADS* variants and *FADS1* and *FADS2* gene expression affect protein expression or LC-PUFA biosynthesis, as changes in gene expression may not translate to changes in enzyme activity. A major strength of the current study is use of large-scale published data to explore the relationship between genetics and *FADS1* and *FADS2* gene expression in many tissues that are difficult to ascertain. However, because the study leverages gene expression measured in tissues collected over a 24-hour window following death, it is possible that the measured *FADS1* and *FADS2* expression levels may not accurately reflect expression levels *in vivo*.

In conclusion, our study for the first time examines the impact of genetic variants in and near the *FADS* gene cluster on *FADS1* and *FADS2* expression in 44 tissues. The analysis revealed that 27 tissues contained significant cis-eQTLs for *FADS1* or *FADS2*, with several tissues containing cis-eQTLs for both. For those tissues containing both, *FADS1* expression and *FADS2* expression were associated with eQTLs in opposite directions. Future studies will be necessary to determine the molecular mechanisms leading to opposite effects of eQTLs on *FADS1* and FADS2, and their ultimate impact on LC-PUFA levels and related molecular and clinical phenotypes.

## Supporting information

S1 Fig Regulatory and genomic features surrounding eQTLs for *FADS1* and *FADS2*. Linkage disequilibrium (r^2^) is shown (top panel) for 32 eQTLs for both FADS1 and FADS2 which had evidence supporting potential functional effects on gene expression (from ENCODE and RegulomeDB). The bottom panel shows the genomic location of eQTLs on chromosome 11, as well as nearby genes, histone marks indicative of regulatory elements (H3K27ac), transcription factor binding sites, DNase clusters, and other predicted regulatory features (from ChromHMM) such as promoters (red), enhancers (orange), and insulators (blue). eQTLs with high prediction scores for functional effects from RegulomeDB (score = 1) are also indicated, as well as variants previously identified as genome-wide significant loci for GWAS investigated traits.
(PDF)

S1 Table 73 SNPs were significantly (FDR<0.05, p≤ 8.30x10-5) associated with both expression of *FADS1* and *FADS2* in same tissue from GTEx. (XLSX)

S2 Table Evidence for function for 73 eQTLs significantly (FDR<0.05) associated with both *FADS1* and *FADS2* in same tissues from GTEx. (XLSX)

S3 Table rs174848 (G vs C) associations (FDR<0.05) with both *FADS1* and *FADS2* in the same tissues from GTEx. (XLSX)
