# Supplementary figures and images for: Tissue-specific impact of FADS cluster variants on FADS1 and FADS2 gene expression
Source: PLoS One. 2018 Mar 28;13(3):e0194610. doi: 10.1371/journal.pone.0194610 (PMC5874031; doi:10.1371/journal.pone.0194610)

# S1 Figure

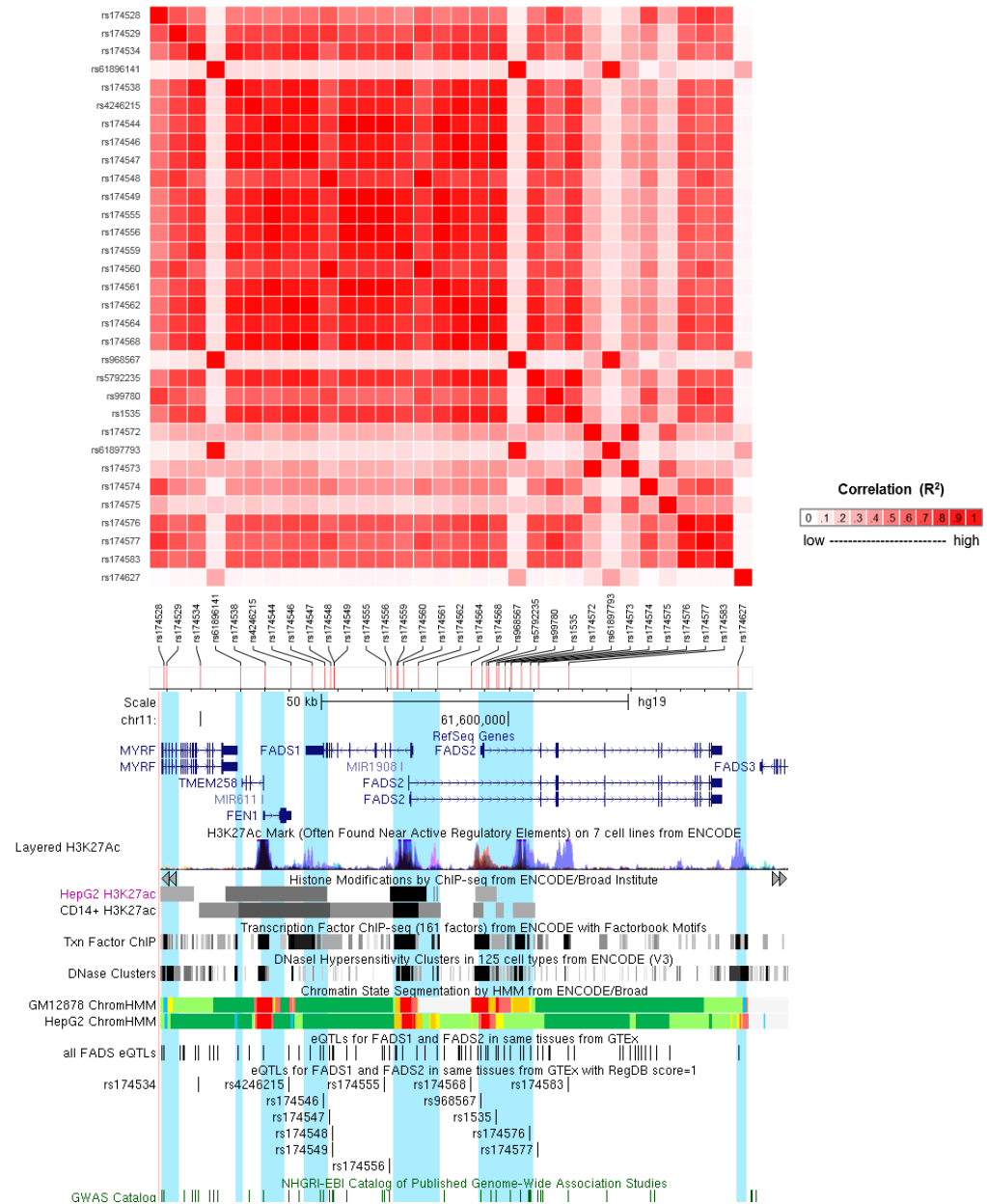

Supplement: S1 Fig — Linkage disequilibrium (r2) is shown (top panel) for 32 eQTLs for both FADS1 and FADS2 which had evidence supporting potential functional effects on gene expression (from ENCODE and RegulomeDB). The bottom panel shows the genomic location of eQTLs on chromosome 11, as well as nearby genes, histone marks indicative of regulatory elements (H3K27ac), transcription factor binding sites, DNase clusters, and other predicted regulatory features (from ChromHMM) such as promoters (red), enhancers (orange), and insulators (blue). eQTLs with high prediction scores for functional effects from RegulomeDB (score = 1) are also indicated, as well as variants previously identified as genome-wide significant loci for GWAS investigated traits. (PDF) [file pone.0194610.s001.pdf]
